# Digital gangrene associated with anticentromere antibodies: a case report

**DOI:** 10.1186/1752-1947-4-189

**Published:** 2010-06-22

**Authors:** Lauren Bolster, Regina M Taylor-Gjevre, Bindu Nair, John A Gjevre

**Affiliations:** 1 Department of Internal Medicine, University of Saskatchewan, Saskatoon, SK, Canada; 2 Division of Rheumatology, Department of Internal Medicine, University of Saskatchewan, Saskatoon, SK, Canada; 3 Division of Respirology, Department of Internal Medicine, University of Saskatchewan, Saskatoon, SK, Canada

## Abstract

**Introduction:**

Anticentromere antibodies have been associated with peripheral vascular occlusive disease, most frequently accompanied by sclerodactyly in the context of a connective tissue disorder. We report a case of digital gangrene with no other clinical associations except positive anticentromere antibodies.

**Case presentation:**

Our patient, a 53-year-old Caucasian woman, non-smoker, presented with progressive pain and blackening of the distal right third finger over the preceding five weeks. No sclerodactyly was evident. She was anticentromere antibody positive at greater than 100 U/mL. Angiography revealed diffuse distal vasculopathy in both upper extremities. Other investigations were unremarkable.

**Conclusions:**

It is rare for anticentromere antibody-associated digital necrosis to develop without concomitant sclerodactyly. However, this patient's case illustrates the need to consider an autoimmune contribution to the pathogenesis of digital ischemia even in the absence of a recognizable connective tissue disease.

## Introduction

The presence of anticentromere antibodies (ACA) is most commonly associated with limited scleroderma. To a lesser extent, ACA have been reported in other disorders including: Raynaud's syndrome, Raynaud's phenomenon associated with sclerodactyly, primary biliary cirrhosis (PBC), and Sjogren's syndrome [1-5]. Patients with circulating ACA associated with limited scleroderma or sclerodactyly have been reported to be at increased risk of significant peripheral vascular occlusive disease [6,7]. We report the case of a 53-year-old woman presenting with digital gangrene and a positive ACA without other features of connective tissue disease.

## Case presentation

A 53-year-old Canadian Caucasian woman, who was a clerical worker, presented to her family doctor with a five week history of progressive pain and black discoloration of the distal right third finger. She was initiated on acetylsalicylic acid and warfarin and referred to a regional tertiary care hospital.

Her past medical history included depression and a diagnosis of Wolfe Parkinson White (WPW) syndrome, treated since childhood with verapamil. She was taking no other medications. She has never smoked and denied a history of Raynaud's type changes in her digits. Her connective tissue disease review of systems was also otherwise unremarkable.

**Figure 1:**
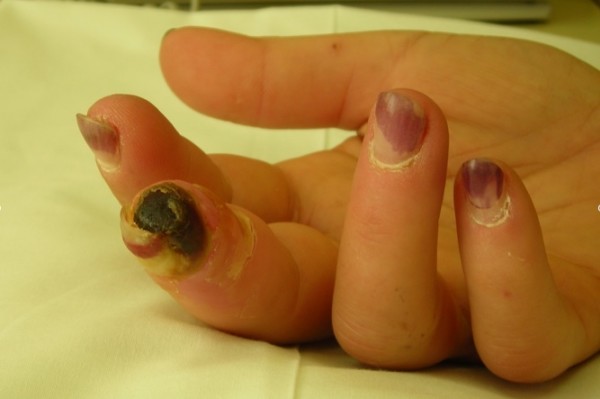
**A photograph of the symptomatic hand demonstrating digital gangrene**. The fingernails are discolored from the presence of residual 'gel-nails'.

On examination in the emergency room, there was obvious digital necrosis of the distal right third finger with an adjacent area of pale swollen tissue with ulceration (Figure 1). Allen's test was abnormal with poor refill bilaterally. Capillaroscopic examination of the periungal regions did not reveal dilated capillary loops. No peripheral bruits were audible. A teleangiectasia lesion was evident on the fifth digit. No other skin changes, specifically sclerodactyly, were present. She was admitted to hospital for further investigations and consultation with vascular specialists.

**Figure 2:**
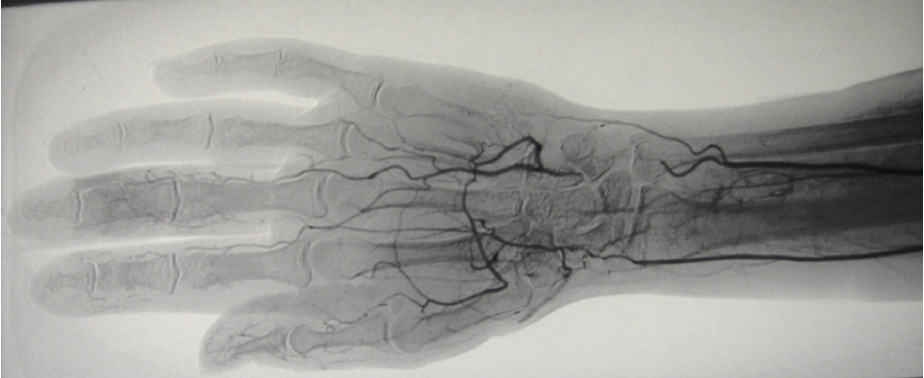
**Angiographic image from the right distal upper extremity demonstrating poor distal flow**.

**Figure 3:**
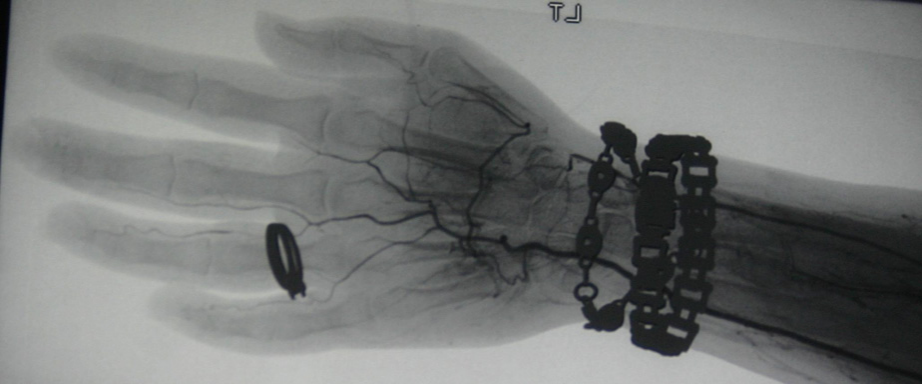
**Angiographic image from the left distal upper extremity demonstrating poor distal flow**.

An angiogram revealed evidence of a bilateral obliterative vasculopathic process (Figures 2 and 3). Radiographs of the hands did not reveal any bony abnormality. Further investigations revealed a positive antinuclear antibody with titer > 1280 and anticentromere specificity. ACA were confirmed by enzyme-linked immunosorbent assay (ELISA) at greater than 100 U/mL. Anti-double stranded DNA, anti-Sjogrens Syndrome A, anti-Sjogrens Syndrome B and anti-ribonucleoprotein antibodies (anti-SSA, anti-SSB, anti-RNP), anti-Sm, anti-Scl-70, antineutrophil cytoplasmic antibodies, anticardiolipin antibodies, cryoglobulins, C3, C4, C-reactive protein, complete blood count, electrolytes, creatinine, hepatic transaminases, alkaline phosphatase and urinalysis were all normal or negative. Associated underlying pathology including cardiopulmonary, gastrointestinal and renal involvement were excluded through cardiology consultation, chest radiograph, echocardiogram, pulmonary function testing, high-resolution computerized tomography (CT) of the chest, 24 hour urine for creatinine clearance, serum chemistry and urinalysis, barium swallow, and CT abdomen and pelvis.

In hospital she was initiated on clopidogrel bisulfate, pentoxifylline, topical nitropaste, a two week trial of prednisone, a seven day course of clindamycin and morphine for pain control. Nifedipine was later initiated as an out-patient. Gradually over the next two months the necrosis resolved with minimal tissue loss at the digit tip. She continues to be followed in the rheumatology out-patient clinic with periodic evaluations for potential evolution of connective tissue disease and in cardiology clinic for follow-up of her WPW.

## Discussion

The association of ACA with peripheral vasculopathy and digital necrosis has been well recognized in the systemic sclerosis population [8]. This association has also been reported in patients whose disease is on the edge of the systemic sclerosis spectrum with Raynaud's and sclerodactyly alone [1,6]. However, it is most unusual for ACA positive patients without concomitant sclerodactyly to develop digital necrosis [8,9].

In our review of the published literature (Table 1), we observed the majority of reported patients had pre-existing Raynaud's phenomenon recognized well prior to the advent of digital necrosis. Other vascular risk factors including smoking, malignancy or previous thermal injury had also been identified in many of these cases. Our patient is the only case we are aware of in which the digital necrosis presented in isolation.

**Table 1:** ACA associated digital necrosis without sclerodactyly: a case comparison

Report	Age (years)	Sex	Pre-existing Raynaud's	Raynaud's duration	Region affected	Co-morbidities	Smoker
**Leon-Perez**[20]	3 cases (39-62)	M	Yes (× 3)	NR	NR	Past frostbite (× 2), HTN (× 2)	Yes (× 3)
							
**Barr**[21]	67	M	Possible	NR	Fingers	CAD, CAGB	No
							
**Takahashi**[10]	60	F	Yes	3 months	Fingers, toes	NR	NR
	74	F	Yes	54 years	Fingers	NR	NR
	79	F	Yes	30 years	Toes	CVA	NR
							
**Picillo**[22]	34	F	Yes	12 years	Fingers, toe	None	Yes
							
**Sachsenberg-Studer**[11]	43	F	Yes	2 years	Fingers	None	Yes
	73	F	Yes	12 years	Fingers	HTN	Never
	84	F	Yes	Unknown	Fingers, toes	HTN	Never
	86	F	Yes	Unknown	Fingers, toe	None	Never
							
**Brown**[23]	87	F	No		Fingers	CHF, HTN	Never
							
**El Mahou**[24]	72	M	Yes	NR	Fingers, toes	SCC	Ex-smoker
							
**Current case**	53	F	No		Finger	WPW	Never

CABG, coronary artery bypass graft; CAD, coronary artery disease; CVA, cerebrovascular accident; HTN, hypertension; NR, not reported; SCC: small cell lung cancer.

It has been postulated that ACA, rather than being a marker antibody may have a direct pathogenic role in vascular endothelial injury [10,11]. It has been observed that human dermal endothelial cells (HDEC) exposed to sera containing ACA demonstrate increased apoptosis and altered gene expression. These include increased expression of genes linked to apoptosis and development of fibrosis, as well as diminished expression of angiogenesis promoting genes [12]. Sera containing ACA has also been shown to have activity against human umbilical vein endothelial cells [13].

Another point of interest in this patient's history is the coexistence of WPW. In this case, the WPW diagnosis dates back to childhood, and is unlikely to be related to the current presentation. However, development of WPW has been recently reported in a patient with scleroderma siné scleroderma [14]. Gross abnormalities in the conducting system have been demonstrated in systemic sclerosis patients via electrophysiologic testing [15]. It has been hypothesized, that myocardial fibrosis may provide substrate necessary for reentrant tachycardia [16-18]. Potentially anti-endothelial activity in ACA positive sera could influence the development of myocardial fibrosis.

In summary, we present a case of a 53-year-old woman with possible early scleroderma [19] who presents with a gangrenous digit, occlusive vasculopathy and a positive ACA, without sclerodactyly, a previous history of Raynaud's phenomenon, or other stigmata of connective tissue disease.

## Conclusions

Our patient's case illustrates the need to consider an autoimmune contribution to pathogenesis of digital ischemia even in the absence of a recognizable connective tissue disease. Furthermore, digital ischemia may be the presenting feature or initial manifestation of an underlying evolving connective tissue disease.

## Consent

Written informed consent was obtained from the patient for publication of this case report and any accompanying images. A copy of the written consent is available for review by the Editor-in-Chief of this journal.

## Competing interests

The authors declare that they have no competing interests.

## Authors' contributions

LB, RTG, BN and JG all analyzed and interpreted aspects of the clinical data associated with this patient's presentation and implications of the same. LB and RTG reviewed the literature and wrote the manuscript. All authors participated in revising and approving the final manuscript.
